# Anesthetic management of a severely obese patient (body mass index 70.1 kg/m^2^) undergoing giant ovarian tumor resection: a case report

**DOI:** 10.1186/s13256-022-03383-x

**Published:** 2022-04-26

**Authors:** Shoko Yamochi, Mao Kinoshita, Teiji Sawa

**Affiliations:** Department of Anesthesiology, Kyoto Prefectural University of Medicine, Kyoto, 602-8566 Japan

**Keywords:** Severe obesity, Giant ovarian tumor, Nasal high flow cannula, Reexpansion pulmonary edema

## Abstract

**Background:**

Giant ovarian tumors are rarely seen with severe obesity. There are few reports of perioperative management of giant ovarian tumors and severe obesity. Here, we report the perioperative management of physiological changes in massive intraabdominal tumors in a patient with severe obesity.

**Case presentation:**

A 46-year-old Japanese woman (height 166 cm, weight 193.2 kg; body mass index 70.1 kg/m^2^) was scheduled to undergo laparotomy for a giant ovarian tumor. The patient was placed in the ramp position. Preoxygenation was performed using a high-flow nasal cannula, and awake tracheal intubation was performed using a video laryngoscope. Mechanical ventilation using a limited tidal volume with moderate positive end-expiratory pressure was applied during the surgical procedure. The aspiration speed for 15 L of tumor aspirate was set to under 1 L/minute, and the possibility of reexpansion pulmonary edema was foreseen by conventional monitoring.

**Conclusions:**

We successfully completed anesthetic management in a patient with concomitant severe obesity and giant ovarian tumors.

## Introduction

Obesity is associated with a variety of complications in general anesthesia, including apnea, hypoventilation [1], and difficulties in intubation [2]. In addition, giant ovarian tumors are very rare in current medical practice, and anesthesiologists are expected be familiar with the physiological changes caused by large tumors. Furthermore, changes in respiratory and circulatory dynamics associated with airway maintenance and large intraabdominal tumors that occur during perioperative complications in patients with both diseases are not well understood. Here, we report a case of anesthetic management and intraoperative changes in respiratory and circulatory dynamics associated with severe obesity and giant ovarian tumors.

## Case presentation

**Fig. 1:**
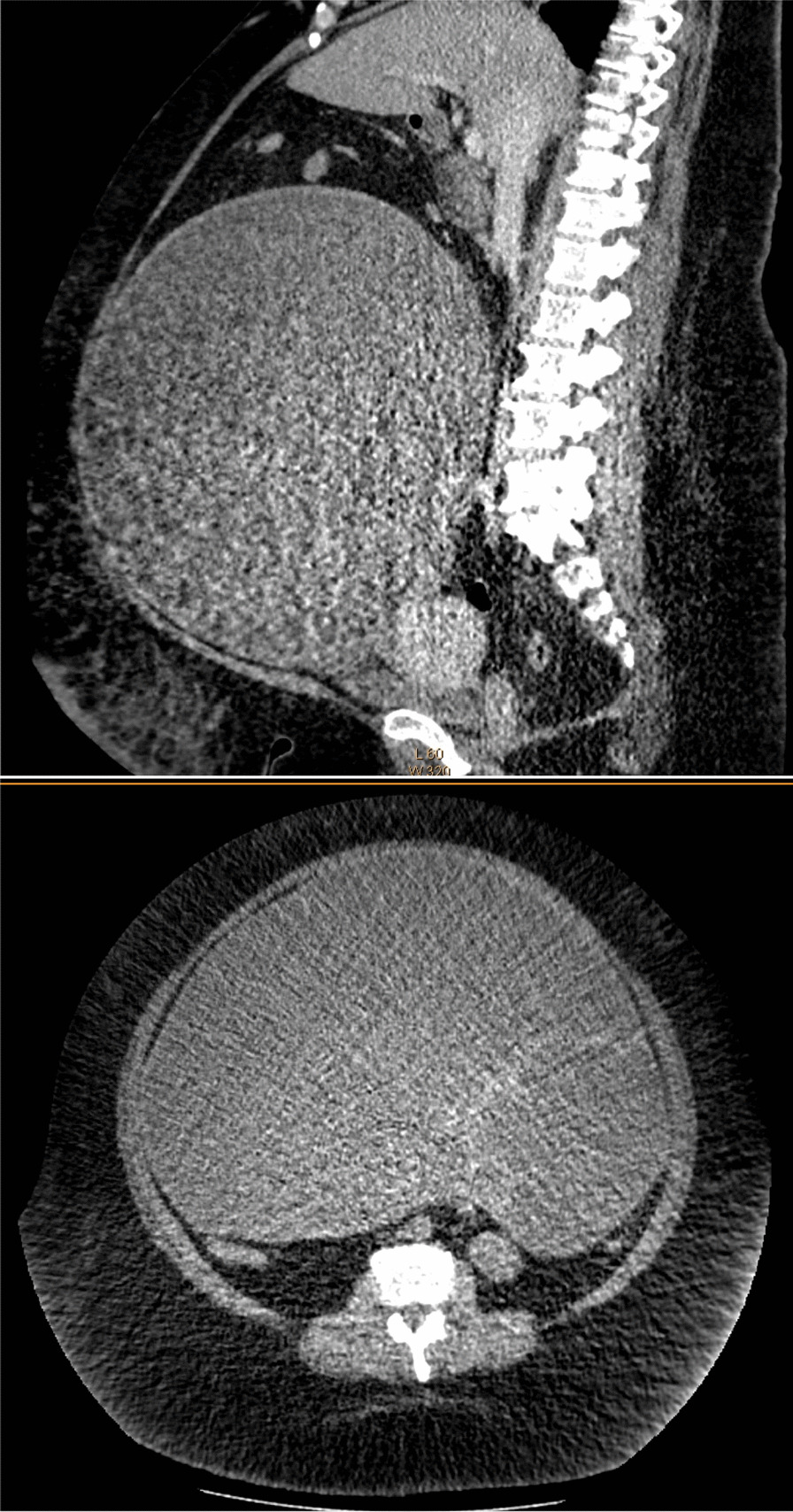
Preoperative abdominal CT shows a giant cystic tumor. Sagittal plane and transverse plane

A 46-year-old Japanese woman (height 166 cm, weight 193.2 kg; body mass index 70.1 kg/m^2^) was scheduled to undergo laparotomy for a giant ovarian tumor. Abdominal computed tomography showed a tensed 35-cm cystic mass that arose from the left ovary and occupied the entire abdomen (Fig. 1). Preoperative pulmonary function tests indicated a restrictive impairment. The patient had no obstructive sleep apnea; therefore, bilevel positive airway pressure was not required. However, she was assessed as bedridden and had a below 2 metabolic equivalent of tasks because she could manage to eat and use the toilet in a sitting position. We predicted difficulty in airway management due to Mallampati III, severe obesity, limited thyromental distance, age (46 years), and thickness of the neck [3]. Acid reduction prophylaxis was not administered the night prior to induction of general anesthesia.

**Fig. 2:**
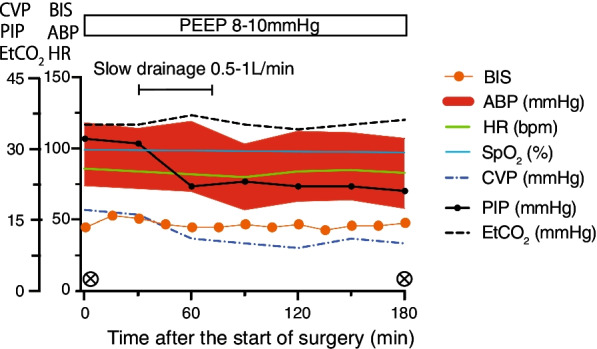
Clinical course before and after tumor removal, anesthetic chart. *BIS* bispectral index, *ABP* arterial blood pressure, *HR* heart rate, *SpO*_*2*_ peripheral blood oxygen saturation, *CVP* central venous pressure, *PIP* peak inspiratory pressure, *EtCO*_*2*_ end-tidal CO_2_, *PEEP* positive end-expiratory pressure

During the induction of anesthesia, in consideration of her discomfort, the patient was placed in the ramp position and preoxygenated with a high-flow nasal cannula (HFNC) at 30 L/minute at an inspired oxygen fraction (FiO_2_) of 1.0. After spraying about 4 ml 2% lidocaine into the pharyngolarynx of the patient, a videolaryngoscope (McGRATH MAC^®^, Aircraft Medical, UK) was inserted while the patient was conscious. Topical airway block was not selected. The patient did not complain of pain, and the glottis could be observed visually. A tracheal tube with inner diameter of 7.0 mm was subsequently intubated. In addition to peripheral insertion of a central venous catheter before surgery, the invasive arterial pressure measurement and peripheral venous path were secured under ultrasonic guidance. Intraoperative anesthesia was maintained by continuous infusion of desflurane using the bispectral index (BIS) target (MAC 1.0); rocuronium bromide was added intermittently to obtain a clinically adequate depth of anesthesia, and remifentanil was administered continuously at 0.1–0.3 μg/kg/minute (ideal body weight 60 kg). After induction of anesthesia, the FiO_2_ was 0.4. The tidal volume (TV) was set at 8 ml/kg predicted body weight and positive end-expiratory pressure (PEEP) of 8–10 mmH_2_O. After an abdominal incision, a total of 15,750 ml of tumor content was carefully aspirated at under 1 L/minute. With aspiration, airway pressure and central venous pressure decreased markedly, and blood pressure stabilized without catecholamine in the infusion load (Fig. 2). Various drugs have been terminated with the completion of surgery, the patient awoke 15 min after discontinuing inhaled agents. After surgery, the patient was extubated with HFNC at 30 L/minute and FiO_2_ of 1.0. The patient’s respiratory condition after extubation was stable, and she was returned to the intensive care unit (ICU) under administration of 6 L/minute of oxygen. Wound infiltration anesthesia was performed in the operative field with 60 ml levobupivacaine (0.25%) because postoperative pain control, epidural anesthesia, and abdominal trunk nerve block were judged to be difficult to perform by prescan using ultrasonic equipment. The operation time was 266 minutes, and the anesthesia time was 404 minutes. The patient lost a small amount of blood, and fluid volume was 1550 ml. The patient was discharged from the ICU on postoperative day 38. Her discharge from the hospital was delayed due to catheter-related infections, diet, weight control, adjustment of antihypertensive and diuretic medications, rehabilitation, and pressure ulcers.

## Discussion

We performed a preoperative evaluation of airway maintenance in a patient with severe obesity, and found that it is possible to safely introduce anesthesia by combining awake tracheal intubation and HFNC. It was also performed at the time of extubation. We were able to perform perioperative management without any complications in response to changes in respiratory and circulatory dynamics associated with the patient’s giant ovarian tumor resection and severe obesity.

Airway management in patients with severe obesity and giant ovarian tumors has been discussed from the respective viewpoints. As a result, the patient was placed in the ramp position, with preoxygenation being performed under the use of HFNC, and awake tracheal intubation being performed using a video laryngoscope. Obese patients develop hypoxemia within 2–4 minute after apnea, even with adequate preoxygenation [4–6]. In this case, severe obesity and intubation difficulties during preoperative airway evaluation were anticipated. Previous studies have shown that head-up [7, 8] or beach-chair [9] positioning improves safe apnea time before the occurrence of significant hypoxemia. Furthermore, NHFC is effective for increasing apnea time under general anesthesia in obese patients and preventing a decrease in peripheral blood oxygen saturation (SpO_2_) [10]. Our patient had a giant ovarian tumor, which might have caused aspiration due to increased abdominal pressure at the time of anesthesia induction. Since there is a possibility of aspiration due to increased abdominal pressure caused by a giant ovarian tumor at the time of induction of anesthesia, induction in lateral decubitus position [11], intubation under consciousness, and induction after suction of ovarian contents under consciousness [12] have been reported. Acid reduction prophylaxis should have been considered for use in this case with material risk factors [13]. In our case, there was a risk of aspiration caused by increased abdominal pressure due to the giant ovarian tumor and severe obesity. Moreover, we believe that combining awake tracheal intubation with NHFC makes airway management safer for severe obesity and giant ovarian tumors. Topical airway block may be advantageous for awake tracheal intubation, but no one could perform it.

When anesthesia with muscle relaxation is introduced after preoxygenation with 100% O_2_, the end-expiratory lung volume is further reduced by about 50% when a PEEP of 5 cmH_2_O is used after the start of ventilation [14]. Thus, the main mechanism of impaired gas exchange in obese patients is shunting (atelectasis). During invasive ventilation, obese patients are more prone to lung collapse and require higher PEEP to avoid it [15]. In this case, we chose to use pressure-assisted ventilation and were able to manage the patient without pressure trauma resulting from increased airway pressure due to positive pressure ventilation.

We were able to perform perioperative management in response to changes in the respiratory and circulatory dynamics associated with tumor resection in a patient with a giant ovarian tumor. The aspiration speed of a total of 15 L of tumor was set to under 1 L/minute, and the possibility of reexpansion pulmonary edema was foreseen by conventional monitoring.

Preoperative aspiration of the tumor is useful in preventing rapid hemodynamic changes and reexpansion of pulmonary edema during the induction of general anesthesia [16]. Intraoperative drainage is often performed at a slow rate of 0.5–1 L/minute [17, 18]. Furthermore, there have been reports of the use of FloTrack^®^ sensors [19], central veins, and transesophageal echocardiography monitors to control the circulatory system. Although reexpansion pulmonary edema might have developed, hypoxia suggestive of reexpansion pulmonary edema did not occur during or after ovarian aspiration. In our case, we gradually reduced the TV to prevent pulmonary edema. During the operation, the patient’s airway pressure and CVP decreased, and her blood pressure stabilized. The possibility of reexpansion pulmonary edema (RPE) due to a rapid decrease in pulmonary thoracic compliance and right heart failure due to the release of pressure on the inferior vena cava before and after tumor resection was foreseen, and efforts were made to stabilize respiration and circulatory dynamics. Peritoneal dissemination of tumor cells was suspected by percutaneous puncture. Alternatively, a double-balloon catheter could have been used preoperatively [13]. It was found that drainage under local anesthesia was difficult because of severe obesity.

It is possible to safely carry out anesthesia introduction and extubation by using HFNC in airway maintenance during anesthesia management in patients with severe obesity. We were able to perform perioperative management in response to changes in circulatory dynamics associated with tumor resection in a patient with a giant ovarian tumor. Furthermore, it has been reported that there are fewer perioperative complications in robot-assisted root surgery than in open surgery, and anesthetic management of obese patients in robotic surgery is also predicted to increase [20].

## Conclusions

Anesthetic management of giant ovarian tumor extraction in a severely obese patient was performed. Our report suggests that, for patients with both of these rare diseases, a combination of conscious intubation and NHFC is necessary, and perioperative management is required to respond to changes in the slow rate of tumor aspiration, mechanical ventilation, and circulatory dynamics in severely obese patients before and after removal of the giant ovarian tumor.

## Data Availability

Not applicable.

## Abbreviations

HFNC: High-flow nasal cannula
FiO_2_: Inspired oxygen fraction
BIS: Bispectral index
TV: Tidal volume
PEEP: Positive end-expiratory pressure
RPE: Reexpansion pulmonary edema
ICU: Intensive care unit
SpO_2_: Peripheral blood oxygen saturation
